# Mr. T. Holmes on John Hunter

**Published:** 1893-10-07

**Authors:** 


The Hospital

An Institutional Journal of

Science, Medicine, Nursing, and Philanthropy.

*For Hospitals, Asylums, Infirmaries, Dispensaries, Medical Practitioners, Students, and Nurses.*

Vol. XV.—No. 367.] [Oct. 7, 1893.

### Mr. T. Holmes on John Hunter.

Reading slowly and carefully Mr. Timothy Holmes' address to the St. George's medical students, on John Hunter, one cannot help asking why Dr. Samuel Smiles has never essayed the task of writing a popular life of the great naturalist-philosopher-surgeon? Mr. Holmes is a Hunter enthusiast, and he did well in his old age to open the winter session of his old hospital with a lime-light presentation of Hunter and his work. It will rather startle the practitioner who has but a traditional knowledge of Hunter to be told that "he occupies a place above the level of any other man who ever practiced any branch of medicine." But this is what Mr. Holmes affirms of him; and, reflection makes one think, with good reason.

Many youthful students will be glad to learn that Hunter was not born with a scalpel in his hand and an inherited physiological treatise in his brain. He was born of the farming class, and probably acquired much of his taste for natural knowledge, real knowledge, that is, by working in the fields in his boyhood. Mr. Holmes tells us that Hunter "never took anything for granted, but tested everything by his own experience." That is a very common habit among those who cultivate the soil intelligently, and as admirable as it is common. It is to be feared that this habit is by no means so common among those who live entirely in the world of books. But it is of all ha bits theone which most makes for scientific and practical progress, and, to the student or the doctor who is at all above the level of the mere reading machine, it is indispensable. We must do real and actual things as well as fill our minds with ideas and statements of fact. If it be said that the modern student or practitioner has not the opportunities of Hunter, the answer is that the student has the dissecting room and the physiological laboratory, and that the practitioner has the bedside. He who has the practical temper of Hunter will find opportunities every day of his life.

But let us return to the judgment pronounced by Mr. Holmes, that Hunter "occupies a place above the level of any other man who ever practised any branch of medicine." The student or practitioner who really loves medicine cannot but experience a good deal of curiosity concerning a man of this measure of elevation. There have been many famous names in physic in many countries, from Hippocrates downwards. It is something for us in this country to have produced the greatest medical man in the world in John Hunter, as well as the greatest poet in Shakespeare, the greatest novelist in Scott, the greatest naturalist in Darwin, the greatest expositor of sociological science in Herbert Spencer, and a host of other greatest names in their several fields of activity. Possibly Mr. Holmes' claim for Hunter will not be allowed to pass altogether without challenge, although the more it is considered the more difficult it will be found to discover any medical man throughout the ages the nature and extent of whose work entitles him to take full and undisputed rank with our British John Hunter.

Mr. Holmes founds his claim for Hunter chiefly on this, that "he was the first who practised and taught surgery as a branch of the science of life." That is to say, Hunter set himself upon the gigantic task of thoroughly knowing and understanding living creatures in every detail of the structure and function of the whole living organism, and of every part of it. To him a human being or an animal was not a mechanism merely, it was a living thing; and the "life," with its "how," its "why," and its "wherefore," was the principal fact about it. Now the merest tyro in physiology knows what a vast, what a difficult, what an almost impossible task Hunter thus set himself He worked at the problem of "life" for half a century, and left it unsolved. The President of the British Association told us but the other day that the problem is unsolved still.

Hunter was a perfectly heroic worker. Thirty years he devoted to the study of the blood. When he was long past middle age, and in the poorest of health, with a permanently diseased heart, he rose, Mr. Holmes tells us, with the sun in the summer, and long before dawn in the winter, and toiled in the dissecting-room from four or five o'clock until his early breakfast at eight. Between eight and four he pursued the ordinary avocations of the practising surgeon. At four he dined, and afterwards slept for an hour. The evening, which most of us devote to rest and recreation, he gave up to still more work, continuing steadfastly at his scientific pursuits until midnight. All this we admire, though few of us imitate it; and it is certainly well that we should not. Many of us it would drive mad, and thus make all our efforts absolutely fruitless and futile. For if a man is to do really good work in science he must preserve a vigorous and capable brain. There is not one man in a million who could work as Hunter did without the absolute destruction of his brain as a thinking organ. Hunter did not destroy his brain, for he did not live long enough. He destroyed his life, however, and that, next to the destruction of the brain, will be admitted to be the most serious of all catastrophes which can happen to a man.

What one would like medical students to think of in connection with Hunter is that he attacked the most difficult of all the problems which nature presents to us because the business of his life lay among those problems. Other surgeons had practised surgery and had gained fame and wealth by it without troubling themselves to explore the profound mysteries of the "life" which animated the living subjects of their work. Other physicians had treated the "blood" of their patients in a thousand different ways without being impelled by the distressing consciousness of their own ignorance to investigate the true nature and possibilities of the "blood." Other pathologists had noticed, and probably wondered at, the phenomena of "inflammation" without thinking of devoting days and nights, months and years, a whole lifetime of often painful and often misunderstood toil, to the elucidation of its manifold and wonderful secrets. The merit of John Hunter, as Mr. Holmes says, lay no doubt in the fact that he was "the first who practised and taught surgery as a branch of the science of life." But the prior merit of recognising that previous medicine and previous surgery had hardly so much as attempted to understand the elementary facts of the life they so light-heartedly dealt with, or the still more elementary facts which constitute the processes of those diseases which it is the business of medicine to treat, was a merit which can only be appreciated by recognising that he was almost the first man in the world's history to perceive the nature of the problems to be solved, as he was certainly the first who made a systematic and, in many respects, successful attempt to solve them.

Every biologist works at "life" to day; every physiologist investigates the "blood," which is in so many senses "the life"; every pathologist devotes his energies to the obtaining of a clear vision of the whole process of "inflammation," and to the clear exposition of the same. These are the common work of biological science in its widest sense. But John Hunter was the pioneer who showed the modern world the way; and it is nothing short of wonderful that of all the workers of the present century hardly one has penetrated further than Hunter into the dark and difficult region into which he carried his daring, his philosophical, and his successful investigations.

Undoubtedly John Hunter was a hero, and Mr. Holmes has been very successful in convincing us of the fact. The students who heard him can never again be as if they had not heard him. Hunter's image, the image of the tireless and true worker, will be in their minds to stimulate or to reproach. This is Hunter's year, the hundredth anniversary of his death. The Hunterian Society celebrates his centenary within the present month. A monument is spoken of. If the great public knew half as much about John Hunter as Mr. Holmes can tell them, a noble monument, worthy of the occasion and the country, would speedily be reared. We trust Mr. Holmes will let the great world, as well as the world of medicine, have the pleasure and the advantage of studying his picture of the life and work of so rare a doctor, philosopher, and man.

